# 

**Published:** 1885-11

**Authors:** 


					﻿
O	jM	!	9 r £
■ & jS	a W- 5 Jfe x. *	%. «?’
i&^XkiP i^Ft 3Mfe rSa W a»A uSssai^ Hr^tS^Ti -SXfS» '

’ |&*■«»	.-
dUIJR
' w ,* v,/. -’’mw \
'‘iulwrintiiiii: |tno« }'. ir in A'lnnKt;
/» inub <innk s, %
F. E. DANIEL, M. D., Editor and Publisher, AUSTIN, TEXAS '
hi’uivE ro.v aoix-KM-ixs, i‘f’ixtE$, xusrfo,	'
				

